# Analysis of DNA Methylation Status in Bodily Fluids for Early Detection of Cancer

**DOI:** 10.3390/ijms18040735

**Published:** 2017-03-30

**Authors:** Keigo Yokoi, Keishi Yamashita, Masahiko Watanabe

**Affiliations:** Department of Surgery, Kitasato University School of Medicine, Kitasato, 1-15-1, Minami-ku, Sagamihara, Kanagawa 252-0374, Japan; butterdog@woody.ocn.ne.jp (K.Yo.); keishi23@med.kitasato-u.ac.jp (K.Ya.)

**Keywords:** methylation, cancer, screening, bodily fluid

## Abstract

Epigenetic alterations by promoter DNA hypermethylation and gene silencing in cancer have been reported over the past few decades. DNA hypermethylation has great potential to serve as a screening marker, a prognostic marker, and a therapeutic surveillance marker in cancer clinics. Some bodily fluids, such as stool or urine, were obtainable without any invasion to the body. Thus, such bodily fluids were suitable samples for high throughput cancer surveillance. Analyzing the methylation status of bodily fluids around the cancer tissue may, additionally, lead to the early detection of cancer, because several genes in cancer tissues are reported to be cancer-specifically hypermethylated. Recently, several studies that analyzed the methylation status of DNA in bodily fluids were conducted, and some of the results have potential for future development and further clinical use. In fact, a stool DNA test was approved by the U.S. Food and Drug Administration (FDA) for the screening of colorectal cancer. Another promising methylation marker has been identified in various bodily fluids for several cancers. We reviewed studies that analyzed DNA methylation in bodily fluids as a less-invasive cancer screening.

## 1. Introduction

In prior decades, many studies have reported epigenetic aberrations in cancer. DNA hypermethylation of the promoter region of specific genes are a major epigenetic change, where some of the cancer-specific methylation genes are tumor suppressor genes. They could be used as molecular markers for the early detection of cancer, prognostic markers, and as therapeutic surveillance markers in cancer therapy [1,2,3,4,5].

Several methods for cancer screening, such as fecal occult blood test (FOBT) in colorectal cancer (CRC), prostate specific antigens (PSA) in prostate cancer, and urine cytology in bladder cancer, are applied clinically. However, most of these methods need improvements due to insufficient sensitivity or specificity. For the purpose of improving these weak points, more invasive methods, such as a colonoscopy or cystoscopy, are used; however, they are not acceptable for high throughput screening due to their invasiveness, technical difficulties, and expensive costs. More sensitive and specific, as well as less invasive methods, with sufficient cost–performance effectiveness, are highly expected to detect cancer cells at an early stage.

In recent years, there has been great progress in the analysis of circulating tumor DNA (ctDNA) in blood. Several studies have shown that the blood of patients with cancer contains cell-free DNA, which shows cancer-related molecular changes [6,7,8]. The origin of ctDNA was not only from circulating tumor cells. The amount of ctDNA was much larger than expected if its origins were lyses from circulating tumor cell [9]. Moreover, Bettegowda et al. [10] reported that ctDNA was often present in patients without detectable circulating tumor cells. These facts suggest another origin of ctDNA. Today, many studies suggest that the main origin of ctDNA is apoptotic and necrotic tumor cells [9,11,12,13]. Several studies have shown the usability of ctDNA, not only for cancer detection, but also for tumor monitoring, prognosis prediction, and early detection of recurrence [8,10,14,15,16,17]. From this point of view, ctDNA analysis demonstrates its usability in dynamic monitoring procedures because ctDNA can be repeatedly obtained, even after the primary tumor has been resected. As the first screening tool for cancer detection, ctDNA analysis is not fully appropriate; the method required to detect cancer-derived DNA alterations from ctDNA in serum or plasma uses highly technique-intensive tools, such as digital polymerase chain reaction (PCR) or next generation sequencers, in which the detection level is up to 1/100,000, while prevalent tools, such as TaqMan PCR, can reach detection levels of 1/1000 [3,18]. Indeed, such advanced methods cannot be performed in many laboratories around the world at present. Furthermore, as many excellent review articles describing ctDNA in blood have already been made available [11,19,20], we will not include them in this review.

There have been several studies, using different methods, which reported cancer specific DNA methylation in bodily fluids around the primary tumor (stool of CRC patients, urine of bladder cancer patients, etc.). Some of the results were very promising and have been approved in standard clinics. Detection of DNA methylation in bodily fluids using classical methods such as quantitative methylation specific PCR, has remained a promising theme. In this article, we reviewed studies that analyzed DNA methylation in less-invasively-obtainable bodily fluids, such as saliva, sputum, stool, and urine; however, we did not include blood. All studies we reviewed focus on promoter hypermethylation, but not the methylation of other genomic regions.

## 2. Methods Used in Methylation Analyses

### 2.1. Sodium Bisulfite Treatment

**Figure 1:**
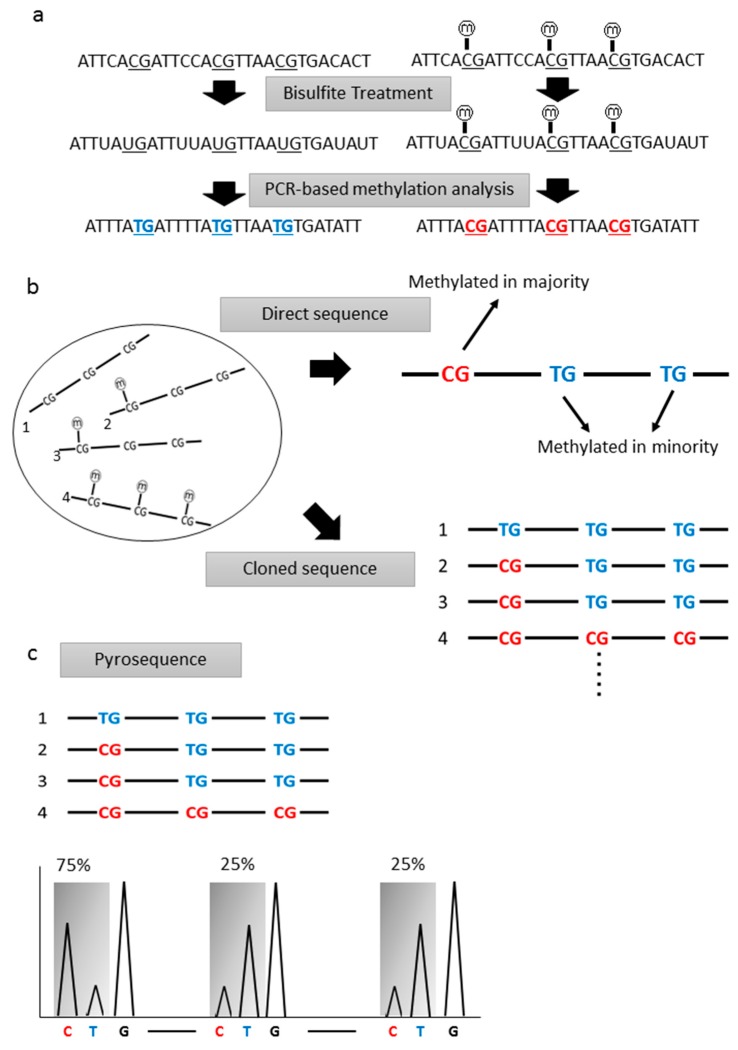
(**a**) A schema of bisulfite treatment of the sample DNA. Unmethylated cytosines were converted to uracil; (**b**) The difference between direct sequence and cloned sequence analyses. Average information of methylation status of each CpG site could be obtained by direct sequence and exact information of each single molecule about each CpG site could be obtained by cloned sequence analysis; (**c**) A schema of pyrosequence analysis. The ratio of methylated molecules could be analyzed using pyrosequence analysis.

Most of the studies we reviewed used PCR-based techniques for methylation analyses. Methylated or unmethylated cytosines were not distinguished by DNA polymerase, thus, the information on epigenetic changes in DNA is lost through the PCR process. Therefore, some modification should be done to distinguish methylated or unmethylated cytosines. By using the bisulfite treatment technique, unmethylated cytosines were converted to uracil, while the methylated cytosines remained unchanged (Figure 1a). If this technique is performed under appropriate conditions, about 99% of unmethylated cytosines are expected to be converted to uracil [21,22]. During subsequent PCR, uracil residues are replaced to thymine residues. Whether the original cytosines were methylated or unmethylated could be analyzed after bisulfite treatment using each of the PCR-based techniques.

### 2.2. Bisulfite Sequence Analyses

Sequence analyses of bisulfite-treated DNA are the simplest method to analyze the methylation status of individual CpG sites. This method was first performed by Frommer et al. [23]. Bisulfite-treated DNA samples are amplified with PCR, and the PCR products are sequenced directly, or sequenced after cloning procedures. Direct sequence analyses of PCR products can determine the average figures of individual CpG sites, while cloned sequence analyses can obtain information from each specific molecule, regarding whether a CpG site is methylated or unmethylated (Figure 1b). For these reasons, direct sequence analyses are performed to screen the major propensity of the DNA methylation status, and cloned sequence analyses are performed for the sake an accurate confirmation of methylation status, even down to single molecules. Quantitative analyses cannot be done via direct sequence analyses, however, and cloned sequence analyses require too much time and money. Thus, for cancer screening, bisulfite sequence analysis is not an appropriate method.

### 2.3. Pyrosequencing

Pyrosequencing analysis is a broadly-used method for quantitative methylation analysis. Bisulfite-treated DNA was amplified by PCR and analyzed using a pyrosequencer. Using this method, each CpG site was quantitatively analyzed for its methylation status (Figure 1c). This method, however, can only analyze a small range of CpG sites, and also requires a cloning procedure. Furthermore, its throughput is lower than the methylation-specific PCR method, and, thus, is not fully appropriate for cancer screening.

### 2.4. Methylation-Specific Polymerase Chain Reaction (PCR)

**Figure 2:**
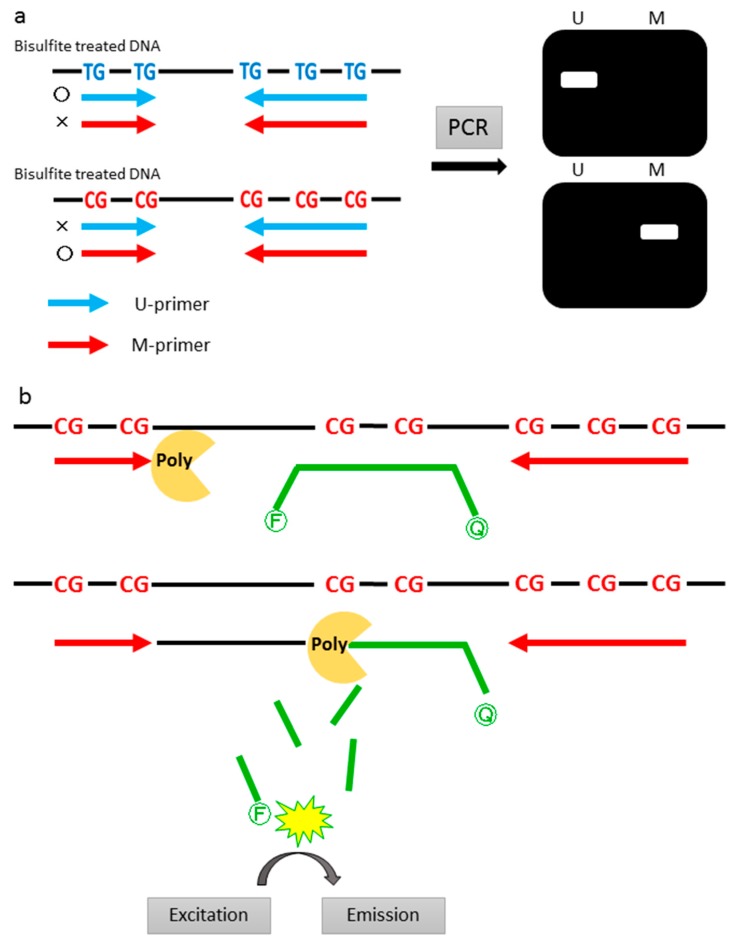
(**a**) A schema of methylation specific PCR. U-primer and M-primer was desigend for each sequence. U, Unmethylated; M, Methylated; (**b**) A schema of quantitative methylation specific PCR. A fluorescent dye and a quencher labelled hybridization probe was desigend between the 2 primers. Fluorescent dye emits its fluorescence when the DNA polymerase cleaved the fluorescent dye from the probe. F, Fluorescent dye; Q, Quencher; Poly, DNA Polymerase.

Methylation-specific PCR (MSP) is the most common method in early studies of methylation analyses. MSP was first reported in 1996 by Herman et al. [24]. This method requires U-primer (primes designed to recognize unmethylated CpGs) and M-primer (primers designed to recognize methylated CpGs) (Figure 2a). M-primers contain several CpG sites (usually one to three in each primer sequence) and, thus, a high specificity for methylation is achieved. Although these are not quantitative methods, high throughput qualitative analyses can be done with a higher sensitivity. Herman et al. [24] developed this method and they reported that MSP could detect methylated templates with a sensitivity of 0.1% in a background of unmethylated templates. However, this conventional MSP has the problem of relatively-frequent, false positive results, especially when performed using a large numbers of PCR cycles [21].

### 2.5. Quantitative Methylation Specific PCR (qMSP)

In the recent studies we reviewed, most of the studies used qMSP as a method of methylation analysis; thus, this method could be called the “gold standard”. Among the quantitative methods, MethyLight, which uses a TaqMan hybridization probe in addition to conventional MSP, is the most common qMSP technique [25]. With the MethyLight technology, sequence discrimination can be done by designing the primers and probe (which can be referred to as Southern hybridization-containing PCR, to further increase its specificity compared to conventional PCR) to contain CpG sites of interest (Figure 2b). The M-primer and U-primer are used as forward and reverse primers, respectively; M-probes (probes designed to recognize methylated CpGs) and U-probes (probes designed to recognize unmethylated CpGs) are then designed. Theoretically, the combination of these primers and probes will design eight sequence variants (2 × 2 × 2) within one sequence. Significant methylation information can be obtained by analyzing, both, fully methylated and fully unmethylated sequences. Quantification of methylation status is calculated from the ratio between the values of these two sequences (fully methylated and unmethylated). Most of the studies reviewed that performed qMSP used a combination of fully methylated sequences (M-primer and M-probe). Quantification can also be done by calculating the ratio between methylated sequences and reference genes (β-actin etc.). With this technique, MSP becomes more specific and no more electrophoresis is required. Furthermore, with the quantitative analysis of methylation status using qMSP, we can select any sensitivity desired in cancer screening.

### 2.6. Methyl BEAMing

**Figure 3:**
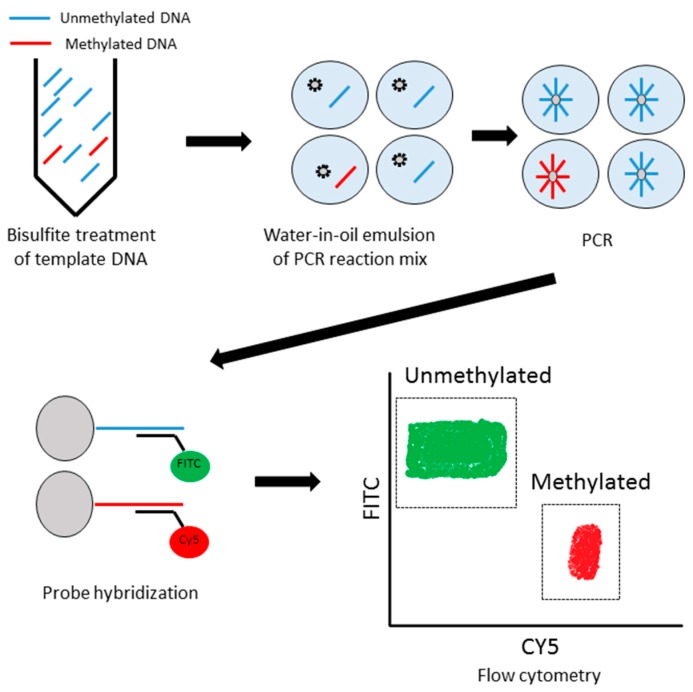
A schema of Methyl-BEAMing. Template DNA were amplified in water-in-oil emulsion by digital PCR. Methylation status was analyzed by a flow cytometer after methylation-specific probe hybridization.

Diel et al. [8] reported on a sensitive assay, called BEAMing, for the detection of mutated ctDNA. The name “BEAMing” was derived from its principal components: Beads, emulsion, amplification, and magnetics (Figure 3). Methyl BEAMing is a modified technique of BEAMing, used for methylation analyses. The PCR primers are designed to amplify methylated and unmethylated templates. PCR amplification of individual DNA molecules takes place within aqueous nanocompartments, suspended in a continuous oil phase. Each aqueous nanocompartment contains the DNA polymerase, template DNA, primers, and beads. When the PCR reaction occurs in each of the compartments, the PCR product binds to the bead so that each bead ends up with thousands of PCR products. After PCR, the beads are collected by breaking the emulsion and the fluorescent probe, which hybridizes specifically to either methylated- or unmethylated-derived sequences on the beads. Li et al. [26] used cyanine dye 5 (Cy5)-labeled probes for methylated sequences, and fluorescein isothiocyanate (FITC)-labeled probes for unmethylated sequences. Beads are analyzed with a flow cytometer.

## 3. Use of Bodily Fluids for Cancer Detection

### 3.1. Saliva

Methylation status of salivary DNA is performed for early detection of head and neck squamous cell carcinoma (HNSCC). Several genes have been analyzed for their promoter hypermethylation. Among the genes, *EDNRB* was the most focused gene for its diagnostic power; however, the specificity was lower than expected.

Saliva is a bodily fluid that can be easily and non-invasively obtained without difficult processes. Saliva has been used for early detection of HNSCCs (Table 1 and Table S1) [27,28,29,30,31,32,33,34]. All reports we reviewed used salivary DNA obtained from a salivary wash (an amount from 10 to 25 mL). A study of the detection of HNSCC using salivary DNA methylation was first reported by Rosas et al. [27] in 2001. Saliva was collected from 30 HNSCC patients and another 30 healthy controls. The methylation status of *p16*, *MGMT*, and *DAPK* genes were analyzed using MSP. At least one gene was hypermethylated in 56.0% of the tumor tissues and 36.6% of the saliva of HNSCC patients. In sixty-five percent of the patients whose tumor tissues were hypermethylated, hypermethylation could be successfully detected in their salivary DNA. In contrast, only one case was hypermethylated in healthy controls. As a result, the sensitivity and specificity of the study was 36.6% and 96.6%, respectively. Righini et al. [28] performed similar analyses, but added another three genes (*TIMP3*, *CDH1*, *RASSF1A*). The sensitivity and specificity of the study were 78% and 100%, respectively. Adding the candidate genes of hypermethylation, they could improve the sensitivity of the study. Carvalho et al. [34] analyzed several gene panels of which the sensitivity and specificity were 22–35% and 90–97%, respectively, by the combination of 13 genes. The association with hypermethylation of *EDNRB* in saliva and HNSCC was analyzed in several studies [29,30,33]. Demokan et al. [30] analyzed hypermethylation of the *EDNRB* gene in HNSCC tissues and saliva. Tumor-specific hypermethylation of *EDNRB* was reported in the study, and the saliva from patients with HNSCC showed frequent hypermethylation. *EDNRB* hypermethylation of saliva was used for the detection of HNSCC with a sensitivity of 67.6% and a specificity of 93.2%. Comparing the other genes reported, *EDNRB* shows a higher sensitivity and specificity for detecting HNSCC. However, the prospective study was conducted by the same group, and the specificity was lower than expected (51%).

### 3.2. Sputum

The methylation status of sputum was analyzed for the detection of lung cancer. Hypermethylation of *RASSF1A* has been analyzed, from early studies to recent studies. Most of the studies containing the *RASSF1A* gene among the analyzed genes resulted in good sensitivity and specificity; thus, hypermethylation of the *RASSF1A* gene might be a promising biomarker for lung cancer detection.

Sputum is broadly used for the detection of lung cancer. Lung cancer is the leading cause of cancer deaths in the world. According to the National Comprehensive Cancer Network (NCCN) guidelines, the first method used for the screening of lung cancer for high risk cohorts is a baseline low-dose computed tomography (CT) [35]. Classically, cytology of sputum has been performed for the purpose of lung cancer diagnosis. The use of sputum is noninvasive and inexpensive compared to a CT scan; however, the sensitivity and specificity of the diagnosis of lung cancer is reported to be 66% and 99% [36], respectively.

In the past few decades, molecular approaches to detect lung cancer, using sputum, have been reported (Table 2 and Table S2) [37,38,39,40,41,42,43,44,45,46,47,48,49,50]. Honorio et al. [38] analyzed promoter hypermethylation of the *RASSF1A* gene in sputum in 2003. The sensitivity was 50% (4/8) for small cell lung cancer (SCLC) and 21% (5/24) for non-small cell lung cancer (NSCLC). Belinsky et al. [43] analyzed hypermethylation of the *p16*, *MGMT*, *RASSF1A*, *DAPK*, *HCAD*, *PAX5α*, *PAX5β*, and *GATA5* genes in sputum and serum using MSP. In their study, they showed that the sensitivity of sputum for the detection of lung cancer was higher than that of serum. The positive predictive value increased to 86% with a panel of the top four genes (*p16*, *DAPK*, *PAX5β*, and *GATA5*) in sputum. Shivapurkar et al. [42] also showed that a combination of several genes could increase the sensitivity. They analyzed four genes (*3-OST-2*, *RASSF1A*, *p16*, and *APC*) according to hypermethylation in sputum. The sensitivity to detecting lung cancer in each gene was 31%, 38%, 23%, and 23%, respectively; however, the sensitivity increased to 54% and 62% with a specificity of 100% by the combination of *3-OST-2* and *RASSF1A* or all of four genes. In recent years, Hubers et al. [47] have produced several reports regarding sputum hypermethylation for lung cancer diagnosis; their first report was published in 2014 [48]. In 2014, they analyzed the hypermethylation of three genes (*RASSF1A*, *APC*, and *cytoglobin*) in relatively large cohorts. Among three genes, *RASSF1A* showed the best results to discriminate lung cancer cases from control cases. Its sensitivity and specificity, in both sets, were 41–52% and 94–96%, respectively. Considering that these results were derived from just one gene, *RASSF1A* might have great potential as a diagnostic biomarker. Furthermore, Hubers et al. [49] validated their results in an independent set with the addition of some genes. In that study, *RASSF1A*, *APC*, *cytoglobin*, *3OST2*, *PRDM14*, *FA19A4*, and *PHACTR3* were analyzed using qMSP. The *RASSF1A* gene, again, showed the best results of sensitivity and specificity among the seven genes (sensitivity of 26.5–42.5%, specificity of 88.3–96.5%). In the most recent reports by Hulbert et al. [50] another set of six genes (*SOX17*, *TAC1*, *HOXA7*, *CDO1*, *HOA9*, and *ZFP42*) was analyzed by using a new extraction method for DNA called methylation-on-beads (MOB), as they thought that a reduction of sensitivity to methylation detection might occur due to technical limitations. Results of the study were surprising, with a sensitivity and specificity in the genes that showed the best results: 86% and 75% (*TAC1*), 63% and 92% (*HOXA7*), and 84% and 88% (*SOX17*). The results of these three genes were comparable to that of *RASSF1A*.

### 3.3. Stool

Methylation analysis in CRC detection has been developed, and the fecal occult blood test demonstrates excellent performance in CRC screening. The fecal DNA test was improved and its ability to be used for cancer screening was compared to the fecal immunochemical test (FIT) in a large cohort; its sensitivity was superior to that of FIT.

Stool has already been widely used for CRC detection in the fecal occult blood test (FOBT). FOBT can be divided into two types: Guaiac FOBT and FIT. Guaiac FOBT has been used for CRC screening since the 1970s, and its usability for reduction in CRC mortality has been proven by several studies [51,52,53]. The sensitivity and specificity of the test is reported to be 31–63% and 92–96% [54,55], respectively. Guaiac FOBT (gFOBT), however, does not positively react specifically to human blood, and so dietary restrictions are necessary for this test. Moreover, gFOBT requires three independent stool samples. On the other hand, FIT detects human hemoglobin using immunological assays. It does not require dietary restrictions and needs a single sample of stool. The sensitivity and specificity of FIT were reported in a recent meta-analysis to be 79% and 94% [56], respectively. Compared to gFOBT, FIT has been performed in several studies, all of which concluded that FIT has a higher sensitivity in detecting colorectal neoplasia [55,57,58].

On the other hand, several of studies reported fecal DNA methylation for the early detection of CRC or adenoma (Table 3 and Table S3) [54,59,60,61,62,63,64,65,66,67,68,69,70,71,72,73,74]. In 2004, Müller et al. [59] analyzed the hypermethylation of several genes and concluded that hypermethylation of the *SFRP2* gene in stool samples can detect CRC with a sensitivity and specificity of 77–90% and 77%, respectively. While the *SFRP2* gene showed excellent sensitivity to detect CRC, its specificity was poor. Lenhard et al. [61] reported that hypermethylation of the *HIC1* gene in stool samples can detect CRC with a sensitivity of 42% and a specificity of 100%. Similarly, Chen et al. [62] reported that hypermethylation of the *vimentin* gene could detect CRC with a sensitivity of 46% and a specificity of 90%. Moreover, hypermethylation of *vimentin* could detect Stage I and II CRC with a sensitivity of 43%. According to these results, analysis of the hypermethylation of single genes potentially has limitations with regard to sensitivity or specificity. From this point of view, Huang et al. [63] analyzed the hypermethylation of the *SFRP2*, *HPP1*, and *MGMT* genes in stool DNA from a large population (52 CRC, 35 benign colorectal disease, and 24 healthy controls). The sensitivity and specificity to detect CRC in the three combined genes was 96.2% and 95.8%, respectively. Among the three genes, *SFRP2* showed the best sensitivity and specificity (94.2% and 95.8%, respectively).

Fecal DNA tests have also been reported as a new screening test for CRC. Stool DNA test 1 (STD-1) and STD-2 are representative. STD-1 consists of a marker panel of 21 point mutations (three on *Kras*, 10 on *APC*, and eight on *p53*). STD-2 consists of three tumor-specific genetic change (*Kras* mutations, scanning of *APC* mutator cluster regions, and methylation of the *vimentin* gene). Ahlquist et al. [54] compared gFOBT, FIT, STD-1, and STD-2 for a screening test of colorectal neoplasm, and reported that, while STD-1 provided no improvement over FIT, STD-2 detected significantly more neoplasms than gFOBT and FIT. Ahlquist et al. [72] developed a next-generation stool DNA test and reported its performance. The test detects four methylated genes *(vimentin*, *NDRG4*, *BMP3*, and *TFPI2*) and mutation of the *kras* gene. Next generation stool tests can detect CRC and adenoma with sensitivities of 85% and 54%, respectively. Moreover, the test had high detection rates for nonmetastatic stages of CRC (87% detection rate for Stage I–III CRCs). Furthermore, important advances in stool DNA tests, such as the use of stabilizing buffers and more discriminating markers, were incorporated. Imperiale et al. [73] reported a new stool DNA test in 2014, and the test (Cologuard^®^ (Exact Science, Madison, WI, USA)) was approved as screening tools for CRC by the U.S. Food and Drug Administration (FDA). The sensitivity for detecting CRC was 92.3% with DNA testing, and 73.8% with FIT. Stool DNA tests could detect CRC significantly better than FIT (*p* = 0.002). Moreover, the DNA test could detect advanced precancerous regions (advanced adenomas or sessile serrated polyps measuring >1 cm) and polyps with high-grade dysplasia (*p* < 0.001 and *p* = 0.004, respectively). The specificity of the DNA test and FIT, however, were 86.6% and 94.9%, respectively, and the number of patients who were excluded from the study was greater in the DNA tests group (*n* = 689) than in the FIT group (*n* = 34). As described above, cancer detection in stool has an advantage compared to other bodily fluids in the detection of cancer.

### 3.4. Urine (For the Detection of Bladder Cancer)

Studies of the analysis of urinary DNA methylation for the detection of bladder cancer have been performed broadly. Despite the numerous studies, none of the promising methylation markers have been found. Recently, urinary tests, combining methylation status and mutation status, were performed in large cohorts.

Urine has also been used to analyze its methylation status for the purpose of the early detection of urinary tract cancers. Classically, cytological examination of urine was performed; however, its sensitivity for low-grade and low-stage cancer was low. For that reason, alternative non-invasive tests have been developed. BTA stat^®^, BTA TRAK^®^, NMP-22^®^, UroVysion™, and ImmunoCyt/uCyt™ are FDA-approved test kits for bladder cancer detection. None of these, however, are used in daily clinical practice because of their low specificity and technical problems [75]. Several other molecular markers have been developed; however, none of the markers were confirmed as promising markers. In this section, studies on methylation analyses using urinary DNA for the detection of bladder cancer are reviewed (Table 4 and Table S4) [76,77,78,79,80,81,82,83,84,85,86,87,88,89,90,91,92,93,94,95,96,97,98,99,100,101,102].

The methylation status of urinary DNA for the detection of bladder cancer was first analyzed in 2002 by Chan et al. [76]. In this study, aberrant methylation in tumor samples was detected in *RARβ2* (87.8%), *DAPK* (58.2%), *E-cadherin* (63.3%), and *p16* (26.5%). Methylation was also analyzed in paired urine samples, of which sensitivities were 45.5%, 68.2%, 59.1%, and 13.6%, respectively.

Hoque et al. [82] analyzed the methylation of urinary DNA in a large cohort (175 for bladder cancer and 94 for age-matched control) using qMSP. They assessed *p16*, *ARF*, *MGMT*, and *GSTP1*; their sensitivities were 45%, 28%, 35%, and 43%, respectively; the specificity was 100%. Despite their low sensitivity to individual genes for the detection of bladder cancer, combined sensitivity (at least one gene) was increased to 69%. Yu et al. [84] also applied multiple gene panels for the detection of bladder cancer using urinary DNA methylation. They analyzed 18 genes, of which sensitivities were 12–58% and they searched for the optimal combination of the genes. A panel of 11 genes (*SALL3*, *CFTR*, *ABCC6*, *HPP1*, *RASSF1A*, *MT1A*, *ALX4*, *CDH13*, *RPRM*, *MINT1*, and *BRCA1*) had the highest sensitivity and a feasible specificity (91.7% and 87.0%, respectively). In this process, the sensitivity and specificity already reached 82.6% and 100% using a combination of four genes (*SALL3*, *CFTR*, *ABCC6*, and *HPP1*), and addition of another set of 11 genes increased the sensitivity to 91.7% in exchange for a decrease in specificity to 87.0%.

In 2010, Renard et al. [86] identified two genes (*TWIST1* and *NID2*) for the early detection of bladder cancer. In their study, they first performed a pharmacologic unmasking microarray to identify the candidate genes for the methylation marker and then they narrowed down the candidates by methylation analysis of bladder cancer tissue samples. Of the 10 genes they identified, they selected the *TWIST1* and *NID2* genes. The panel of two genes was applied to the detection of bladder cancer using urinary DNA methylation analyses. A higher sensitivity and feasible specificity were shown in both the training and validation sets (sensitivity; 88% and 94%, specificity; 94% and 91%, respectively), and, notably, a sensitivity of 80–89% was found for early-stage and low-grade cancer. This study, however, was later validated by two prospective studies that failed to replicate the excellent performance [98,100]. Recently, Dahmcke et al. [102] conducted a prospective study for the detection of bladder cancer in a patient cohort of gross hematuria. In this study, the methylation status of six genes (*SALL3*, *ONECUT2*, *CCNA1*, *BCL2*, *EOMES*, and *VIM*) and the mutation of two genes (*TERT* and *FGFR3*) were used as a test for urinary DNA. Patients with hematuria were concluded in the study (*n* = 475), and all of the patients underwent cystoscopy. Ninety-nine patients were diagnosed with bladder cancer. The sensitivity and specificity of the test were 97% and 77%, respectively, and the area under the curve (AUC) of the test was calculated as 96.3%. The negative predictive value was 99.0%, and, with this value, the author noted that this DNA test could reduce the number of patients who would need to undergo cystoscopy.

### 3.5. Urine (For the Detection of Prostate Cancer)

Contrary to studies on the detection of bladder cancer, the number of urinary DNA methylation studies for prostate cancer is small; however, almost all studies analyzed the methylation status of *GSTP1*. Early studies suggested that *GSTP1* methylation is a promising marker; however, a recent prospective study revealed it to have insufficient specificity.

The methylation status of urine has also been analyzed for the early detection of prostate cancer. The standard tools for early detection of prostate cancer are PSA and digital rectal exam (DRE). Due to its lower specificity in the discrimination of benign prostate hyperplasia (BPH), however, false positive results have been a clinical problem. Only 25% of men with PSA values between 4 and 10 ng/mL have a positive biopsy [103]. If the cut off value was set to 4.1 ng/mL, the sensitivity and specificity were reported to be 20.5% and 93.8% [104], respectively. A new molecular biomarker is still required for the screening of prostate cancer. In this section, studies of methylation analyses of urinary DNA for the early detection of prostate cancer are reviewed (Table 5 and Table S5) [105,106,107,108,109,110,111,112,113,114].

Urinary DNA methylation analysis for the screening of prostate cancer was first reported in 2000 by Goessl et al. [105]. They used the MSP method with a fluorescent labeled primer for methylation analyses. They analyzed the methylation status of GSTP1 in urine, plasma, serum, and ejaculate, of which the sensitivity of urinary GSTP1 methylation was 36% and the specificity was 100%. The same group later analyzed GSTP1 methylation in urine samples, which were obtained after a 15 to 30 s prostate massage [107]. Using this method, the sensitivity of GSTP1 methylation increased to 73%. Moreover, when focusing on T1-2N0M0 patients, the sensitivity increased from 0% (0 of 11 patients) to 68% (15 of 22 patients). Hoque et al. [110] also analyzed urinary GSTP1 methylation using qMSP with eight other genes (*p16*, *ARF*, *MGMT*, *RARβ*, *E-cadherin*, *TIMP3*, *RASSF1A*, and *APC*). The urine samples of age-matched individuals with no history of genitourinary malignancies were used as controls. The sensitivity and specificity of GSTP1 were 48% and 100%, respectively. The panel of four genes (*p16*, *ARF*, *MGMT*, and *GSTP1*) could discriminate prostate cancer with a sensitivity and specificity of 87% and 100%, respectively. In 2008, Venar et al. [114] conducted a multicenter prospective study for patients with PSA levels higher than 2.5 ng/mL. Urinary samples were collected after the DRE procedure. The methylation status of GSTP1, RARβ2, and APC were analyzed. The sensitivity and specificity of three combined genes were 53–55% and 76–80%, respectively. Another prospective study was conducted in 2009 for patients with PSA levels from 2 to 10 ng/mL, which contained 178 prostate cancer patients and 159 noncancerous patients [112]. The sensitivity and specificity of the combination of three genes were 60% and 81%, respectively. The specificities of these studies were lower than expected.

## 4. Conclusions

Several studies were conducted regarding cancer screening via the analysis of methylation in bodily fluids. In CRC screening, a higher sensitivity and specificity of stool DNA tests were reported and they can be used in clinical situations. In other cancers, there were no promising methylation markers that could be used in clinics. Some of the gene candidates, such as *NID2* or *TWIST1* in urinary DNA, seemed to be promising markers, however, these studies are denied in prospective studies [86,98,100].

Most of the studies combined several genes in order to increase sensitivity, as the sensitivity of each gene was generally low. As the number of genes increased, the cost and time needed for the tests also will increase greatly. Simple and low-cost tests are required in cancer screening because it is performed for millions of people. To resolve this problem, a promising single methylation marker, which has a high sensitivity and specificity, needs to be explored.

## Figures and Tables

**Table 1:** Studies of methylation analysis of saliva for the detection of head and neck squamous cell carcinoma.

Author	Year	Method	Prospective Study	Sample Size (Number of Patients)	Gene	Sensitivity (%)	Specificity (%)
Rosas et al. [27]	2001	MSP	No	HNSCC (30) Healthy control (30)	*DAPK*, *MGMT*, *p16*	At least 1 gene (37%)	At least 1 gene (97%)
Righini et al. [28]	2007	MSP	No	HNSCC (60) Non Malignant (30)	*CDH1*, *DAPK*, *MGMT*, *p16*, *RASSF1*, *TIMP3*	At least 1 gene (79%)	At least 1 gene (100%)
Carvalho et al. [34]	2008	qMSP	No	HNSCC (211) Control (527)	*AIM1*, *CCNA1*, *CCND2*, *CDH1*, *DAPK*, *DCC*, *ESR1*, *MGMT*, *MINT1*, *MINT31*, *PGP9.5*, *p16*, *TIMP3*	At least 1 of 4 gene *(31%)*	At least 1 of 4 gene *(90%)*
Demokan et al. [29]	2010	qMSP	No	HNSCC (71) Healthy Control (61)	*EDNRB*, *KIF1A*	*EDRNB* + *KIF1A* (77%)	*EDRNB* + *KIF1A* (93%)
Pattani et al. [30]	2010	qMSP	Yes	Clinically high risk patients Total (191) Malignant (35) Premalignant (43) Benign (113)	*EDNRB*	*EDNRB* (65%)	*EDNRB* (51%)
Carvalho et al. [31]	2011	qMSP	No	HNSCC (61)	*CCNA1*, *DAPK*, *DCC*, *MGMT*, *MINT31*, *p16*, *TIMP3*	At least 1 gene (54%)	
Schussel et al. [33]	2013	qMSP	Yes	Clinically high risk patients HNSCC or dysplasia (48) Benign (113)	*DCC*, *EDNRB*	*EDRNB* + *DCC* + risk classification (75%)	*EDRNB* + *DCC* + risk classification (48%)
Rettori et al. [32]	2013	qMSP	No	HNSCC (146) Healthy control (60)	*CCNA1*, *DAPK*, *DCC*, *MGMT*, *TIMP3*, and other 19 genes	At least 1 gene (55%)	At least 1 gene (76%)

**Table 2:** Studies of methylation analysis of the sputum for the detection of lung cancer.

Author	Year	Method	Prospective Study	Sample Size (Number of Patients)	Gene	Sensitivity (%)	Specificity (%)
Belinsky et al. [37]	1998	MSP	No	LC (7) Smokers (26)	*p16*	*p16* (43%)	*p16* (81%)
Honorio et al. [38]	2003	MSP	No	SCLC (8) NSCLC (24) Chronic Smokers (13)	*RASSF1A*	SCLC; *RASSF1A* (50%) NSCLC; *RASSF1A* (21%) Chronic Smokers; *RASSF1A* (31%)	
Konno et al. [39]	2004	MSP	No	LC (78) None LC (52)	*APC*, *p16*, *RARβ*	*APC* (28%), *p16* (22%), *RARβ* (27%)	*APC* (96%), *p16* (100%), *RARβ* (93%)
Belinsky et al. [41]	2006	Nested MSP	No	LC (98) Healthy Controls (92)	*BETA3*, *DAPK*, *GATA4*, *GATA5*, *HCAD*, *HLHP*, *IGFBP3*, *LAMC2*, *MGMT*, *PAX5α*, *PAX5β*, *p16*, *RASSF1A*, *SFRP1*	*GATA5* (74%), *LAMC2* (72%), SFRP1 (68%)	*GATA5* (74%), *LAMC2* (30%), *SFRP1* (29%)
Cirincione et al. [40]	2006	MSP	No	LC (18) Healthy Controls (smoker) (112)	*p16*, *RARβ2*, *RASSF1A*	At least 1 gene (50%)	At least 1 gene (38%)
Belinsky et al. [43]	2007	MSP	No	LC (Stage III) (72)	*DAPK*, *GATA5*, *HCAD*, *MGMT*, *PAX5α*, *PAX5β*, *p16*, *RASSF1A*	*GATA5* (43%), *MGMT* (32%), *p16* (40%)	
Shivapurkar et al. [42]	2007	qMSP	No	NSCLC (13) Controls without LC (25)	*APC*, *p16*, *RASSF1A*, *HS3ST2*	At least 1 gene (62%)	At least 1 gene (100%)
Shivapurkar et al. [44]	2008	qMSP	No	LC (13) Non Cancer (25)	*CYGB*	*CYGB* (30%)	*CYGB* (100%)
Guzmán et al. [45]	2012	MSP	No	LC (26) COPD (23) Healthy Controls (33)	*CDH1*, *MGMT*, *p16*	LC *CDH1* (35%), *MGMT* (65%), *p16* (73%) COPD *CDH1* (45%), *MGMT* (65%), *p16* (70%) Healthy controls *CDH1* (32%), *MGMT* (6%), *p16* (9%)	
Leng et al. [46]	2012	Nested MSP (cohort1) qMSP (cohort2)	No	Cohort 1 LC (64) Non Cancer (64) Cohort 2 LC (40) Non Cancer (90)	*GATA5*, *PAX5α*, *SULF2*	Cohort 1 *GATA5* (33%), PAX5α (25%), *SULF2* (34%) Cohort 2 *GATA5* (78%), PAX5α (63%), *SULF2* (78%)	Cohort 1 *GATA5* (74%), PAX5α (80%), *SULF2* (75%) Cohort 2 *GATA5* (53%), PAX5α (67%), *SULF2* (45%)
Hubers et al. [48]	2014	qMSP	No	LC (20) COPD (31)	*APC*, *CYGB*, *FAM19A4*, *HS3ST2*, *PHACTR3*, *PRDM14*, *RASSF1A*	*RASSF1A* + *3OST2 (85%)*	*RASSF1A* + *3OST2 (74%)*
Hubers et al. [47]	2014	qMSP	No	Set1 LC (98) None LC (90) Set2 LC (60) none LC (445)	*APC*, *CYGB*, *RASSF1A*	Set1; At least 1 gene (63%) Set2; At least 1 gene (90%)	Set1; At least 1 gene (78%) Set2; At least 1 gene (47%)
Hubers et al. [49]	2015	qMSP	No	Learning set LC (73) none LC (86) Validation set LC (159) none LC (154)	*APC*, *CYGB*, *FA19A4*, *HS3ST2*, *PHACTR3*, *PRDM14*, *RASSF1A*	Learning Set *HS3ST2* + *PHACTR3* + *RASSF1A* (82%) Validation Set *HS3ST2* + *PHACTR3* + *RASSF1A* (79%)	Learning Set *HS3ST2* + *PHACTR3* + *RASSF1A* (66%) Validation Set *HS3ST2* + *PHACTR3* + *RASSF1A* (64%)
Hulbert et al. [50]	2016	qMSP	No	LC (90) none LC (24)	*CDO1*, *HOXA7*, *HOXA9*, *SOX17*, *TAC1*, *ZFP42*	HOXA7 + SOX17 + TAC1 (98%)	*HOXA7* + *SOX17* + *TAC1* (71%)

LC: Lung Cancer; COPD: Chronic Obstructive Pulmonary Disease.

**Table 3:** Studies of methylation analysis of the stool for the detection of colorectal cancer.

Author	Year	Method	Prospective Study	Sample Size (Number of Patients)	Gene	Sensitivity (%)	Specificity (%)
Song et al. [60]	2004	MSP	No	CRC (20) Normal CF (20)	*APC*, *ATM*, *HLTF*, *MGMT*, *hMLH-1*	At least 1 gene (70%)	
Müller et al. [59]	2004	qMSP	No	Training Set CRC (10) Healthy Control (13) Validation Set CRC (13) Healthy Control (13)	*SFRP2*	Training Set; *SFRP2* (90%) Validation Set; *SFRP2* (77%)	Training Set; *SFRP2* (77%) Validation Set; *SFRP2* (77%)
Lenhard et al. [61]	2005	MSP	No	CRC (26) Adenoma (13) Hyperplastic Polyp (9) CIBD (9) Normal control (32)	*HIC1*	CRC; *HIC1* (42%) Adenoma; *HIC1* (31%)	CRC + Adenoma; *HIC1* (98%)
Chen et al. [62]	2005	MSP	No	CRC (94) Normal Control (198)	*VIM*	All Stages; VIM (46%) Stage I and II; *VIM* (43%)	*VIM* (90%)
Huang et al. [63]	2007	MSP	No	CRC (52) Adenoma (21) Hyperplastic Polyp (8) Ulcerative Colitis (6) Healthy Control (24)	*HPP1*, *MGMT*,*SFRP2*	CRC; At least 1 gene (96%) Adenoma; At least 1 gene (71%)	CRC + Adenoma; At least 1 gene (96%)
Zhang et al. [64]	2007	MSP	No	CRC (29) Adenoma (7) Healthy Control (17)	*SFRP1*	CRC + Adenoma; *SFRP1* (89%)	CRC + Adenoma; *SFRP1* (86%)
Wang et al. [65]	2008	qMSP	No	CRC (69) Adenoma (34) Hyperplastic Polyp (26) Healthy Control (30)	*SFRP2*	CRC; *SFRP2* (87%) Adenoma; *SFRP2* (62%) Hyperplastic Polyp; SFRP2 (42%)	CRC + Adenoma; *SFRP2* (93%)
Ahlquist et al. [54]	2008	OBT SDT	Yes	Total (3764) CRC (39) Adenoma (251)	SDT-1 SDT-2	CRC + Adenoma Hemoccult (11%), HemoccultSensa (21%), SDT-1 (20%), SDT-2 (40%)	CRC + Adenoma; Hemoccult (98%), HemoccultSensa (97%), SDT-1 (96%)
Nagasaka et al. [69]	2009	Hi-SA	No	CRC (84) Adenoma (56) Hyperplastic Polyp (12) Without Neoplasms (113) Other Disease (31)	*RASSF2*, *SFRP2*	CRC *RASSF2* (27%), *SFRP2* (31%) Adenoma *RASSF2* (11%), *SFRP2* (25%)	CRC + Adenoma; *RASSF2* (95%), *SFRP2* (92%)
Glöckner et al. [68]	2009	MSP	No	CRC (84) Adenoma (26) CF negative control (87)	*TFPI2*	Training Set CRC; *TFPI2* (89%) Validation Set CRC; *TFPI2* (76%) Adenoma; *TFPI2* (21%)	Training Set CRC; *TFPI2* (79%) Validation Set CRC; *TFPI2* (93%) Adenoma; *TFPI2* (93%)
Hellebrekers et al. [70]	2009	qMSP	No	Set1 CRC (28) Healthy Control (45) Set2 CRC (47) Healthy Controls (30)	*GATA4* *GATA5*	Set1; *GATA4* (71%) Set2; *GATA4* (51%)	Set1; *GATA4* (84%) Set2; *GATA4* (93%)
Melotte et al. [71]	2009	qMSP	No	Training Set CRC (28) CF Negative Control (45) Validation Set CRC (47) CF Negative Control (30)	*NDRG4*	Training Set; *NDRG4* (61%) Validation Set; *NDRG4* (53%)	Training Set; *NDRG4* (93%) Validation Set; *NDRG4* (100%)
Baek et al. [66]	2009	MSP	No	CRC (60) Adenoma (52) CF Negative (37)	*MGMT*, *hMLH1*, *VIM*	CRC; At least 1 gene (75%) Adenoma; At least 1 gene (60%)	CRC + Adenoma; *MGMT* (86%), *hMLH1* (100%), *VIM* (100%)
Kim et al. [67]	2009	qMSP	No	CRC (20) Adenoma (17) CF Normal (15)	*B4GALT*, *OSMR*, *SFRP1*	CRC; *OSMR* + *SFRP* (60%) Adenoma; *OSMR* + *SFRP1* (35%)	CRC + Adenoma; *OSMR* + *SFRP1* (100%)
Ahlquist et al. [72]	2012	SDT	No	CRC (252) Adenoma (133) CF Negative Control (293)	*BMP3*, *NDRG4*, *TFPI2*, *VIM*, *kras* (mutation)	CRC; SDT (85%) Adenoma (>1 cm); SDT (63%)	CRC; SDT (89%) Adenoma (>1 cm); SDT (89%)
Imperiale et al. [73]	2014	SDT	Yes	Total (9989) CRC (65)	*BMP3*, *NDRG4*, *kras* (mutation)	SDT (92%), FIT (74%)	SDT (90%), FIT (96%)
Zhang et al. [74]	2014	MSP	No	CRC (48) Adenoma (35) Hyperplastic Polyp (32) Healthy Control (30)	*SFRP2*, *WIF-1*	CRC; *SFRP2* + *WIF-1* (81%) Adenoma; *SFRP2* + *WIF-1* (81%)	CRC + Adenoma; *SFRP2* + *WIF-1* (97%)

CF: Colon Fiber; CIBD: Chronic Inflammatory Bowel Disease; Hi-SA: High-Sensitivity Assay for Bisulfite DNA; SDT-1: Stool DNA Test-1 (point mutations of *kras*, *APC*, and *p53*); SDT-2: Stool DNA Test-2 (*kras* mutation, *APC* mutator cluster regions, and methylation of *VIM*); OBT: Occult Blood Test.

**Table 4:** Studies of methylation analysis of the urine for the detection of bladder cancer.

Author	Year	Method	Prospective Study	Sample Size (Number of Patients)	Gene	Sensitivity (%)	Specificity (%)
Chan et al. [76]	2002	MSP	No	BC (22) Normal Control (17)	*DAPK*, *E-cadherin*, *p16*, *RARβ*	At least 1 gene (91%)	At least 1 gene (77%)
Chan et al. [77]	2003	MSP	No	BC (14) Normal control (10)	*RASSF1A*	*RASSF1A* (50%)	*RASSF1A* (100%)
Sathyanarayana et al. [79]	2004	MSP	No	BC (71) Bladder Wash (28) Voided Urine (43) None Malignant (6)	*LAMA3*, *LAMB3*, *LAMC2*	At least 1 gene (49%)	At least 1 gene (100%)
Friedrich et al. [78]	2004	qMSP	No	BC (37) Normal Control (20)	*BCL2*, *DAPK*, *TERT*	*BCL2* (65%), *DAPK* (22%), *TERT* (51%)	At least 1 gene (100%)
Dulaimi et al. [80]	2004	MSP	No	BC (45) normal (12) Inflammatory Urinary Disease (9)	*APC*, *p14*, *RASSF1A*	At least 1 gene (87%)	At least 1 gene (100%)
Hoque et al. [82]	2006	qMSP	No	BC (175) Normal Control (94)	*ARF*, *GSTP1*, *MGMT*, *p16*	At least 1 gene (69%)	At least 1 gene (100%)
Urakami S et al. [83]	2006	MSP	No	BC (24) Normal Control (20)	*DKK3*, *SFRP1*, *SFRP2*, *SFRP4*, *SFRP5*, *WIF1*	At least 1 gene (61%)	At least 1 gene (93%)
Yates et al. [81]	2006	qMSP	Yes	BC (35) Benign Control (35) Healthy Volunteer (34)	*APC*, *DAPK*, *E-cadherin*, *GSTP1*, *p14*, *p16*, *RARB*, *RASSF1A*	*APC* + *E-cadherin* + *RASSF1A* (69%)	*APC* + *E-cadherin* + *RASSF1A* (60%)
Yu et al. [84]	2007	MSP	No	BC (132) Normal Control (7) Noncancerous Urinary Lesion (23) Other disease(6)	*ABCC6*, *ALX4*, *BCL2*, *BMP3*, *BRCA1*, *CCNA1*, *CDH13*, *CFTR*, *DRM*, *HPR1*, *ITGA4*, *MINT1*, *MTA1*, *MYOD1*, *RASSF1A*, *RPRM*, *RUNX3*, *SALL3*	Combination of 11 genes (92%)	Combination of 11 genes (87%)
Sun et al. [85]	2009	MSP	No	BC (82) Noncancerous Urinary Lesion (15) Normal Control (5)	*CDH1*, *FANCF*, *LOXL1*, *LOXL4*, *p16*, *SFRP1*, *SOX9*, *TIG1*, *TIMP3*, *XAF1*	*LOXL1* (40%), *SFRP1* (37%), *XAF1* (71%)	*LOXL1* (73%), *SFRP1* (93%), *XAF1* (33%)
Lin et al. [87]	2010	MSP	No	BC (57) Normal Control (20)	*E-cadherin*, *p14*, *p16*, *RASSF1A*	At least 1 gene (83%)	
Renard et al. [86]	2010	qMSP	Yes	Symptomatic patients Training Set BC (48) Normal Control (121) Validation Set BC (35) Normal Control (57)	*NID2*, *TWIST1*	Training Set; *NID2* and *TWIST1* (88%) Validation Set; *NID2* and *TWIST1* (94%)	Training Set; *NID2* and *TWIST1* (94%) Validation Set; *NID2* and *TWIST1* (91%)
Reinert et al. [89]	2011	MS-HRM	No	BC (115) BPH or Bladder Stone (59)	*EOMES*, *HOXA9*, *POU4F2*, *ZNF154*	At least 3 genes (84%)	At least 3 genes (96%)
Eissa et al. [88]	2011	MSP	No	BC (210) Benign Urological Disease (61) Normal Control (49)	*APC*, *RARβ2*	*APC* (60%), *RARβ2* (63%)	*APC* (84%), *RARβ2* (95%)
Chen et al. [91]	2011	qMSP	No	BC (30) None Cancer Control (19)	*DAPK*, *IRF8*, *p14*, *RASSF1A*, *SFRP1*	*IRF8* (57%), *p14* (28%), *SFRP1* (41%)	*IRF8* (95%), *p14* (100%), *SFRP1* (100%)
Vinci et al. [90]	2011	qMSP	Yes	Bladder cancer (108) Control (105) BPH (29) Urinary tract infection (17) Bladder Stone (16) Normal Volunteer(43)	*BCL2*, *DAPK*, *hTERT*	At least 1 gene (79%)	At least 1 gene (90%)
Serizawa et al. [92]	2011	qMSP	No	BC (113) Normal Control (33)	*APC*, *DBC1*, *RARB*, *RASSF1A*, *SFRP1*, *SFRP2*, *SFRP4*, *SFRP5* *FGFR* (mutation), *PIK3CA* (mutation), *RAS* (mutation), *TP53* (mutation)	Total (70%)	Total (94%)
Chung et al. [94]	2011	qMSP	No	BC (128) None Cancer Control (110)	*A2BP1*, *CA10*, *DBC1*, *MYO3A*, *NKX6-2*, *NPTX2*, *PENK*, *SOX11*	*CA10* + *MYO3A* + *NKX6-2* + *SOX11* (81%)	*CA10* + *MYO3A* + *NKX6-2* + *SOX11* (97%)
Costa et al. [93]	2011	qMSP	No	BC (50) RCC (50) PC (50) Healthy Control (48)	*PCDH17*, *TCF21*	BC; *PCDH17* + *TCF21* (60%) RCC; *PCDH17* + *TCF21* (32%) PC; *PCDH17* + *TCF21* (26%)	BC; *PCDH17* + *TCF21* (100%) RCC; *PCDH17* + *TCF21* (100%) PC; *PCDH17* + *TCF21* (100%)
Reinert et al. [95]	2012	qMSP	No	BC (184) BPH or Bladder Stone (35)	*EOMES*, *HOXA9*, *POU4F2*, *TWIST1*, *VIM*, *ZNF154*	*EOMES* (88%), *TWIST1* (88%), *VIM* (89%)	*EOMES* (97%), *TWIST1* (100%), *VIM* (100%)
Chihara et al. [96]	2013	Pyrosequencing	No	BC (73) Healthy Volunteer (18)	Hypermethylation; *HOXA9_1*, *HOXA9_2*, *MYOD*, *SOX1*, *TJP2* Hypomethylation; *CAPG*, *CASP8*, *HLADPA1*, *IFNG*, *RIPK3*, *SPP1*, *VAMP8*	*HOXA9_1* (86%), *HOXA9_2* (86%), *MYOD* (87%), *TJP2* (93%)	*HOXA9_1* (89%), *HOXA9_2* (62%), *MYOD* (88%), *TJP2* (56%)
Abern et al. [98]	2014	qMSP	Yes	Hematuria or on surveillance for prior NMIBC Total (111) BC (24) None Cancer Control (87)	*NID2*, *TWIST1*	*NID2*, *TWIST1* (75%)	*NID2*, *TWIST1* (71%)
Hayashi et al. [97]	2014	qMSP	No	BC (20) Normal Control (20)	*VGF*	*VGF* (40%)	*VGF* (95%)
Fantony et al. [100]	2015	qMSP	Yes	Hematuria or on surveillance for prior NMIBC, or NMIBC treated with BCG Total (209) BC (52) Suspicious of BC (12) Negative for Cystoscopy (145)	*NID2*, *TWIST1*	Believe the positive (67%) Believe the negative (37%)	Believe the positive (61%) Believe the negative (86%)
Yeh et al. [99]	2015	qMSP	No	Training set BC (69) None Cancer Control (28) Test set BC (33) None Cancer Control (28)	*ZNF671*	Training Set; *ZNF671* (42%) Test Set; *ZNF671* (48%)	Training set; *ZNF671* (93%) Test set; *ZNF671* (89%)
Dahmcke et al. [102]	2016	qMSP	Yes	Hematuria Total (475) BC (99) None Cancer Control (376)	*BCL2*, *CCNA1*, *EOMES*, *ONECUT2*, *SALL3*, *VIM*, *FGFR* (mutation), *TERT* (mutation)	Total (97%)	Total (77%)
Roperch et al. [101]	2016	qMSP	No	BC (167) None Cancer Control (105)	*HS3ST2*, *SEPT9*, *SLIT2*, *FGFR3 (mutation)*	Total (Methylation + Mutation) (98%)	Total (Methylation + Mutation) (85%)

BC: Bladder Cancer; RCC: Renal Cell Carcinoma; BPH: Benign Prostate Hyperplasia; NMIBC: Non Muscle Invasive Bladder Cancer; MS-HRM: Methylation Sensitive High Resolution Melting.

**Table 5:** Studies of methylation analysis of the urine for the detection of prostate cancer.

Author	Year	Method	Prospective Study	Sample Size (Number of Patients)	Gene	Sensitivity (%)	Specificity (%)
Goessl et al. [105]	2000	MSP	No	PC (33) BPH (26)	*GSTP1*	*GSTP1* (36%)	*GSTP1* (100%)
Goessl et al. [108]	2001	MSP	No	PC (29) BPH (40)	*GSTP1*	*GSTP1* (77%)	*GSTP1* (97%)
Goessl et al. [107]	2001	MSP	No	PC (40) PIN (7) BPH (45)	*GSTP1*	PC; *GSTP1* (73%) PIN; *GSTP1* (29%)	PC and PIN; *GSTP1* (98%)
Cairns et al. [106]	2001	MSP	No	PC (22)	*GSTP1*	*GSTP1* (27%)	
Jeronimo et al. [109]	2002	MSP qMSP	No	PC (69) BPH (31)	*GSTP1*	qMSP; *GSTP1* (19%) MSP; *GSTP1* (30%)	qMSP and MSP; *GSTP1* (67%)
Hoque et al. [110]	2005	qMSP	No	PC (52) None Cancer Control (91)	*APC*, *ARF*, *E-cadherin*, *GSTP1*, *MGMT*, *p16*, *RARβ2*, *RASSF1A*, *TIMP3*	At least 1 of 4 genes (87%)	At least 1 of 4 genes (100%)
Roupret et al. [111]	2007	qMSP	No	PC (95) None Cancer Control (38)	*APC*, *CDH1*, *DAPK*, *GSTP1*, *MGMT*, *p14*, *p16*, *RARβ2*, *RASSF1A*, *TIMP3*	At least 1 of 4 genes (89%)	At least 1 of 4 genes (89%)
Venar et al. [114]	2008	qMSP	Yes	PSA > 2.5 ng/mL Biopsy Positive (111) Biopsy Negative (123)	*APC*, *GSTP1*, *RARβ2*	Cohort1; At least 1 gene (55%) Cohort2; At least 1 gene (53%)	Cohort1; At least 1 gene (80%) Cohort2; At least 1 gene (76%)
Baden et al. [112]	2009	qMSP	Yes	PSA 2–10 ng/mL PC(178) None Cancer Control (159)	*APC*, *GSTP1*, *RARβ2*	At least 1 Gene (60%)	At least 1 Gene (81%)
Daniunaite et al. [113]	2014	qMSP	No	PC (253) BPH (32)	*GSTP1*, *RARB*, *RASSF1*	*GSTP1* (11%), *RARB* (29%), *RASSF1* (45%)	*GSTP1* (97%), *RARB* (81%), *RASSF1* (84%)

PC: Prostate Cancer; PIN: Prostatic Intraepithelial Neoplasia.

## Supplementary Materials

Supplementary materials can be found at www.mdpi.com/1422-0067/18/4/735/s1.
